# Vaginal Cleansing and Post-Cesarean Infectious Morbidity? Updated Systematic Review and Meta-Analysis of Randomized Trials

**DOI:** 10.3390/biomedicines13061505

**Published:** 2025-06-19

**Authors:** Marco La Verde, Marco Torella, Irene Iavarone, Rossella Molitierno, Antonio Cerillo, Margherita Casillo, Maria Maddalena Marrapodi, Mario Fordellone, Liliana Mariani, Chiara Melito, Barbara Gardella, Mattia Dominoni

**Affiliations:** 1 Department of Woman, Child and General and Specialized Surgery, University of Campania “Luigi Vanvitelli”, 80138 Naples, Italy; marco.torella@unicampania.it (M.T.); ireneiavarone2@gmail.com (I.I.); rossella.molitierno@studenti.unicampania.it (R.M.); antoniocerillo@gmail.com (A.C.); casillo.margherita@gmail.com (M.C.); 2 Pediatric Unit, Department of Woman, Child and General and Specialized Surgery, University of Campania “Luigi Vanvitelli”, 80138 Naples, Italy; mariamaddalena.marrapodi@unicampania.it; 3 Medical Statistics Unit, University of Campania “Luigi Vanvitelli”, 80138 Naples, Italy; mario.fordellone@unicampania.it; 4 Department of Clinical, Surgical, Diagnostic and Paediatric Sciences, University of Pavia, 27100 Pavia, Italy; liliana.mariani01@universitadipavia.it (L.M.); c.melito2808@gmail.com (C.M.); barbara.gardella@gmail.com (B.G.); matti.domino@gmail.com (M.D.); 5 Department of Obstetrics and Gynecology, IRCCS Foundation Policlinico San Matteo, 27100 Pavia, Italy

**Keywords:** cesarean delivery, endometritis, postoperative fever, wound infection, vaginal preparation, post-cesarean endometritis, post-cesarean infections, post-cesarean infectious morbidity

## Abstract

**Background**: Endometritis, maternal fever and wound infection represent the most frequent post-cesarean complications. The aim of the present research was to evaluate the incidence of post-cesarean infections after vaginal cleansing. **Materials and methods**: The databases analyzed were MEDLINE, Scopus, EMBASE, CENTRAL, Google Scholar, Clinicaltrials.gov and the Register of Controlled Trials. No language or geographical restrictions were applied. We included only randomized controlled trials that analyzed various vaginal antiseptic solutions to reduce postpartum endometritis. The terms employed were as follows: vaginal solution, cesarean section, endometritis, wound infection, chlorhexidine, povidone, metronidazole, cetrimide, and pregnancy. The PICO categorization was as follows: P—population: pregnant women; I—intervention: vaginal antiseptic; C—control: hands-off or routine care; O—outcome: post-cesarean endometritis, wound infection and postoperative fever; S—study design: randomized controlled trials. **Results**: A total of 32 articles, including 13,853 participants, were selected. The vaginal cleansing group showed a low incidence of endometritis. The chlorhexidine group had an OR of 0.56 (95% CI 0.45–0.70, *p* = 0.010). The povidone group had an OR of 0.47 (95% CI 0.37–0.59, *p* = 0.002). Considering maternal fever, 2598 patients from 5 studies in the chlorhexidine group were analyzed, alongside 6965 patients from 18 trials in the povidone group. The povidone group presented an Odds ratio of 0.47 (95% CI 0.38–0.57, *p* = 0.0001). A reduction in wound infection incidence was observed in the povidone group (OR = 0.59, 95% CI = 0.42–0.82, *p* < 0.05). **Conclusions**: Vaginal cleansing before cesarean section, particularly with povidone solutions, reduces the incidence of postoperative endometritis and maternal fever.

## 1. Introduction

Cesarean section (CS) is a surgical procedure associated with various complications [1]. Complication rates vary between 6 and 29 percent, dependent upon the study design, definitions and populations [2,3,4]. The most common post-cesarean complications are endometritis (6–27%), maternal fever (5–24%) and wound infection (2–9%) [5]. In comparison to vaginal births, these complications are considerably higher [6]. The implementation of CS antibiotic prophylaxis has reduced endometritis incidence by 75%; however, CS continues to have an important role in postoperative cesarean endometritis development [7,8,9]. The consequences of endometritis are bacteremia, peritonitis, intraabdominal abscess, and sepsis [10]. In addition, seroma and hematoma are wound morbidities [11]. They cause discomfort and prolonged hospitalization for patients [11]. It is hypothesized that abnormal vaginal bacteria flora, which can occur in association with fever, pelvic abscess, and sepsis, is the primary cause [12]. Research on vaginal bacterial alteration after antibiotic prophylaxis is limited [13,14,15]. Over the past two decades, several RCTs have been carried out to explore the association between preoperative vaginal disinfection and postoperative infection [16]. In addition, the National Institute for Health and Care Excellence (NICE) suggests aqueous iodine vaginal preparation before performing CS on a pregnant patient whose delivery is complicated by the preterm rupture of membranes [17]. Aqueous chlorhexidine vaginal preparation can be used if aqueous iodine vaginal preparation is unavailable or not recommended [18]. Different preparations for vaginal cleansing have been suggested to prevent the risk of post-cesarean infection [11]. Aqueous iodine vaginal preparations have also been shown to lower endometritis risk in women with ruptured membranes [18]. Higher efficacy was shown with iodine vaginal formulations [18]. Endometritis prevention solutions based on povidone and chlorhexidine are the most readily available, with the latter most active against anaerobic microorganisms [19,20]. In contrast, alternative solutions provide no a clear benefit in accordance with scientific evidence [21].

Our meta-analysis aims to investigate whether vaginal cleansing alongside prophylactic antibiotics would be a feasible option for decreasing the incidence of post-cesarean endometritis by evaluating data from published randomized controlled trials (RCTs). It is postulated that vaginal cleansing reduces the bacterial load in the vagina and reduces the incidence of postoperative infectious morbidity. As secondary outcomes, we analyze whether the addition of vaginal cleansing may reduce the incidence of maternal fever and wound infection.

## 2. Materials and Methods

### 2.1. Eligibility Criteria and Inclusion Criteria

This systematic review and meta-analysis was performed according to the Preferred Reporting Items for Systematic Reviews and Meta-Analyses [22] and the methods outlined in Mbuagbaw et al. [23]. The research protocol was recorded in the International Prospective Register of Systematic Reviews (PROSPERO) database (CRD42023465294). We evaluated RCTs that aimed to reduce postpartum endometritis using different vaginal antiseptic solutions, comparing them to each other or to a placebo or no treatment, in pregnant patients who had received antibiotics prior to or during the CS. The prespecified primary outcome was endometritis, defined as a maternal temperature > 38 °C (100.4 °F) with uterine tenderness and/or foul-smelling vaginal discharge. Secondary outcomes were wound infection and postoperative fever. Wound infection was defined most often as swelling, erythema, discharge, seroma, hematoma, or disruption of the incision line. Fever was defined as a temperature greater than 38 °C (101.2 °F) at least 24 h after delivery. The PICO categorization was as follows: P—population: pregnant women; I—intervention: vaginal antiseptic; C—control: hands-off or routine care; O—outcome: post-cesarean endometritis, wound infection and postoperative fever; S—study design: randomized controlled trials. We included only studies with pregnant women who had received antibiotic prophylaxis before or during their cesarean section and had received a vaginal antiseptic solution to prevent postpartum endometritis. Quasi-randomized and non-randomized trials, studies in which women did not receive antibiotic prophylaxis, or study interventions that did not target vaginal antisepsis were excluded. Letters, editorials, comments, and opinions were also excluded.

### 2.2. Search Strategy

We conducted an extensive review of the MEDLINE (accessed through PubMed), Scopus, EMBASE and Cochrane Central Register of Controlled Trials (CENTRAL) databases from their origin to 23 January 2024. The search strategy included words from related texts regarding vaginal solutions, cesarean section, endometritis, wound infection, chlorhexidine, povidone, metronidazole, cetrimide, and pregnancy. A filter for randomized controlled trials only was applied to the search results (Appendix A). To reduce publication bias Google scholar was also searched. Moreover, the gray literature (NTIS, PsycEXTRA) was screened to search for the abstracts of international and national conferences. We also reviewed the references of the included studies and the related reviews for additional papers not captured during the original search. No language or geographic location restrictions were applied. Commentaries, letters to the editor, editorials, and reviews were excluded from the review. Other exclusion criteria included the following: quasi-randomized trials and trials without randomization and studies including patients undergoing cesarean section without antibiotic prophylaxis.

### 2.3. Study Selection

The abstracts were systematically evaluated and classified by two authors (I.I. and L.M.) independently. Agreement on possible relevance was accomplished by consensus. The full texts of the selected studies were evaluated by the same two authors, who extracted relevant data regarding the study details and the outcomes of interest in an autonomous manner. Upon consulting a third author (M.L.V.), the authors established a consensus after deliberating on every disagreement. Following the data screening process, the full texts of the selected abstracts were collected. Full texts, titles and abstracts that lacked adequate information according to the inclusion criteria were also acquired. Full-text articles were selected that complied with the inclusion criteria by the two reviewers using a data extraction form.

### 2.4. Data Extraction and Statistical Analysis

The data analysis was performed by R Studio version 4.1.3 (2022-03-10). The data extracted from the included articles for further analysis were as follows: demographic information (title, authors, journal and year), the characteristics of the sample (age, gestational age, delivery number, inclusion and exclusion criteria and number of participants), study-specific parameters (study type, form of application of perineal massage, duration of massage, time of application and use of lubricants), the follow-up and dropout rates of participants, and the results obtained (variables analyzed, instruments used, and results throughout the follow up). The characteristics of the trials and the extracted data were both presented in tables. Non-randomized or quasi-randomized studies were excluded. We also excluded studies if the outcome measures were inconsistent with our criteria or the intervention was not related to antiseptic vaginal wash. The meta-analysis was performed using the random-effects model, because of the observed heterogeneity. For each sub-study, the Odds ratios (OR) with their corresponding 95% confidence intervals (CI) were calculated from the summary data provided. Heterogeneity was assessed using Cochran’s Q test, with a *p*-value < 0.05 considered indicative of significant heterogeneity. Although I^2^ statistics are commonly used to quantify heterogeneity, in our analysis only the Q test was applied, as the reporting of between-study variance was limited in several included studies. Due to the limited number of studies in some subgroup analyses, a formal assessment of publication bias (e.g., funnel plot asymmetry or Egger’s test) was not feasible. Therefore, potential bias remains a limitation. Only complete outcome data were used for the synthesis.

### 2.5. Assessment of Risk of Bias

The assessment of the risk of bias in every study included was conducted in accordance with the standards delineated in the *Cochrane Handbook for Systematic Reviews of Interventions* [24]. Each included trial underwent a critical examination of the following seven domains, as it became apparent that these concerns were associated with biased estimates of the effects of treatments: (1) random sequence generation; (2) allocation concealment; (3) the blinding of participants and personnel; (4) the blinding of outcome assessment; (5) incomplete outcome data; (6) selective reporting, and (7) other bias. The authors classified the evaluation of their judgements as having a “low risk”, a “high risk”, or an “unclear risk” of bias [24]. The risk-of-bias assessment was independently judged by 3 authors (I.I., L.M., C.M.). Disagreement was resolved by discussion with a fourth reviewer (M.L.V.).

## 3. Results

### 3.1. Study Selection

**Figure 1:**
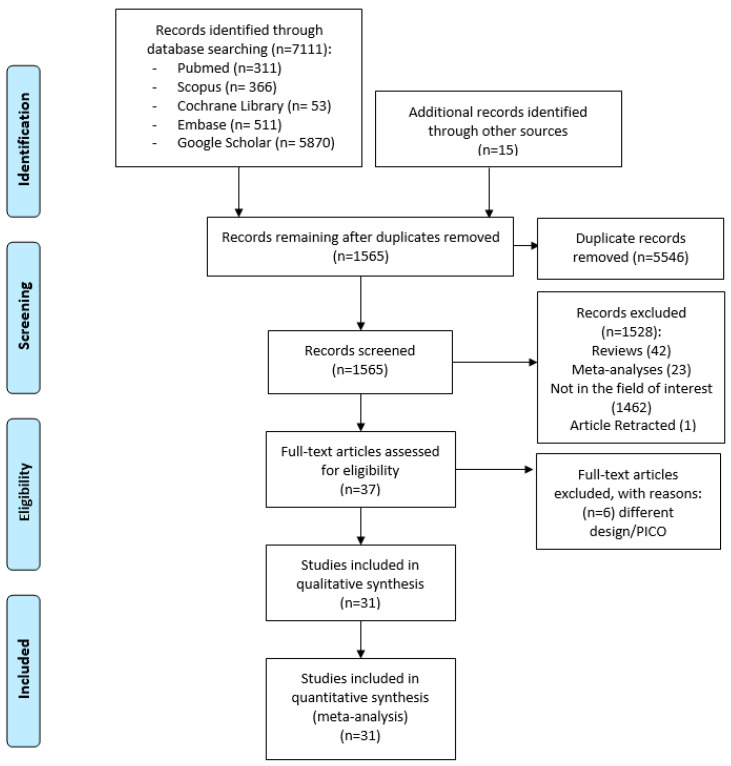
PRISMA flow diagram of the database search from the database origin to 23 January 2024.

Of the 7126 total results identified, 5546 records were duplicates, so 1565 underwent title and abstract screening to see if they met the inclusion criteria (Figure 1). Of these, 38 were then excluded according to the inclusion and exclusion criteria. Of the 37 articles screened, 31 were finally selected (Figure 1). In total, 31 studies, including 13,627 participants, were included in the quantitative synthesis and network meta-analysis (Figure 1) [5,25,26,27,28,29,30,31,32,33,34,35,36,37,38,39,40,41,42,43,44,45,46,47,48,49,50,51,52,53,54].

### 3.2. Study Characteristics

The studies were conducted between 1997 and 2023. All studies included women with a diagnosis of endometritis, defined as a maternal temperature > 38 °C (100.4 °F) associated with uterine tenderness and/or foul-smelling vaginal discharge. A full list of the studies included is reported in Table 1 with the study information (year, country, number of participants, intervention and control groups, primary outcomes). Our meta-analysis included 32 RCTs with a total of 13,853 subjects [5,25,26,27,28,29,30,31,32,33,34,35,36,37,38,39,40,41,42,43,44,45,46,47,48,49,50,51,52,53,54]. A total of 10 studies adopted vaginal cleansing with chlorhexidine [5,25,26,27,28,29,30,31,32,33] and included 6230 patients: 3089 in the treatment group and 3141 subjects in the control group. Different chlorhexidine solution concentrations were adopted: 0.05% [29], 0.20–0.25% [25,26,27], 0.4% [5], 1% [32], 4% [28,31], and 7.5% [33]. A total of 20 studies out of 32 evaluated povidone solution [34,35,36,37,38,39,40,41,42,43,44,45,46,47,48,49,50,51,52] and included 7399 patients: 3638 in the treatment group and 3651 subjects in the control group. The povidone iodine solution was applied at 1% [11,47,52], 5% [41,43,45] or 10% [35,37,38,39,42,44,46,48,50]. Two studies [37,51], in addition to a povidone and a control group, included a third group, in which participants were treated with a saline vaginal solution, for a total of 110 patients. Only one study [53] adopted metronidazole vaginal gel, with 112 patients in the treatment group and 112 in the control group. Vaginal preparation with cetrimide solution was adopted in only one study, with 100 patients in the treatment group and 100 in the control group [54]. The control groups of several studies included no vaginal cleansing or sterile saline solution or placebo vaginal gel.

### 3.3. Risk of Bias of Included Studies

**Figure 2:**
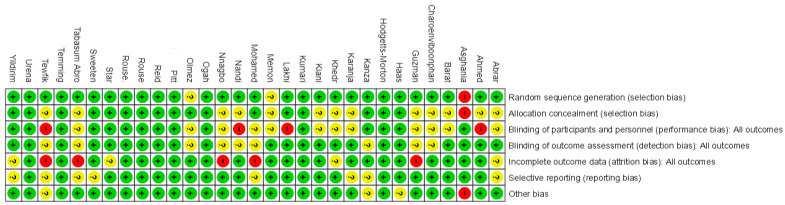
Risk of bias: summary. Green (+) indicates low risk of bias, red (−) indicates high risk of bias, and yellow (?) indicates unclear risk of bias.

**Figure 3:**
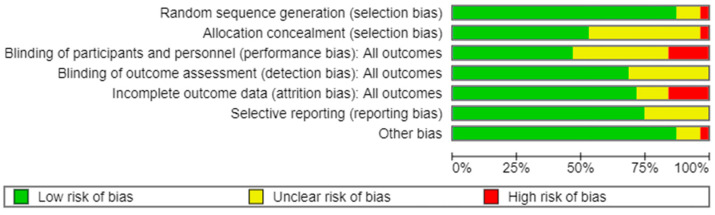
Risk of bias: graph. Green indicates low risk of bias, yellow indicates unclear risk of bias, and red indicates high risk of bias.

Figure 2 displays the methodological quality of each trial, and a summary of the quality of methodologies, expressed in percentages across all trials, is represented in Figure 3. The majority of the research included presented a low risk of bias. Data about random sequence generation were reported for 28 out of 31 trials.

### 3.4. Synthesis of Results

#### 3.4.1. Endometritis

**Figure 4:**
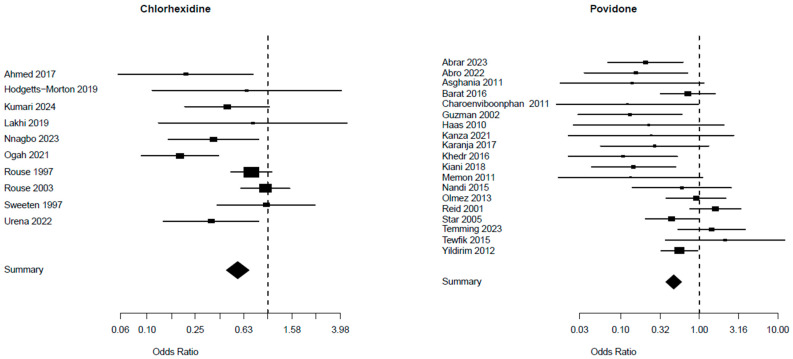
Risk of endometritis from studies [5,25,26,27,28,29,30,31,32,33] for chlorhexidine and [34,35,36,37,38,39,40,41,42,43,44,45,46,47,48,49,50,51,52] for povidone.

A comparison of the risk of endometritis between the two vaginal solutions (chlorhexidine and povidone) and the control group is presented in Table 2: the chlorhexidine group involved 10 trials with a total of 6230 patients, whereas the povidone group included 19 studies with a total of 7173 patients. The chlorhexidine group presented a Mantel–Haenszel Odds ratio of 0.56 (95% CI 0.45–0.70) and a test of heterogeneity value of 21.63, with a *p* = 0.010. The Mantel–Haenszel Odds ratio was 0.47 (95% CI = 0.38–0.60) and the test of heterogeneity value was of 41.00, *p* = 0. 0015, in the povidone group (Figure 4). The I^2^ values for endometritis were 47.3% in the chlorhexidine group and 54.5% in the povidone group (Table 2).

#### 3.4.2. Maternal Fever

**Figure 5:**
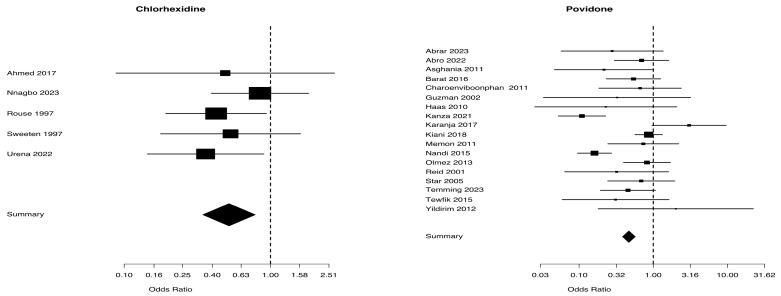
Maternal fever from studies [5,25,27,30,31] for chlaorhexidine and [34,35,36,38,39,40,41,42,43,44,45,46,47,48,49,50,51,52] for povidone.

A total of 2598 patients from five studies were included in the chlorhexidine group, whereas 6965 patients from 18 trials were included in the povidone group, considering the difference in maternal fever between the two groups, as shown in Table 2. The Mantel–Haenszel Odds ratio was 0.52 (95% CI 0.34–0.79) and the test of heterogeneity was especially notable at 2.44, with a *p*-value of 0.656, for the chlorhexidine group. The Mantel–Haenszel Odds ratio was 0.47 (95% CI = 0.38–0.57) and the test of heterogeneity value was of 57.72, *p* = 0.0001, in the povidone group (Figure 5). The I^2^ values for maternal fever were 0% in the chlorhexidine group and 70.5% in the povidone group (Table 2).

#### 3.4.3. Wound Infection

**Figure 6:**
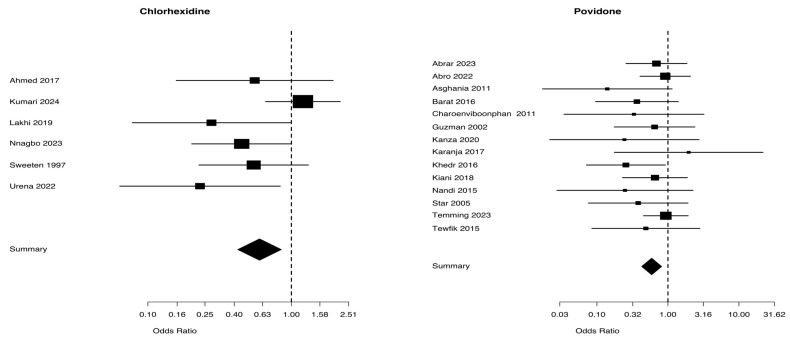
Wound infection from studies [5,25,28,30,31,33] for chlaorhexidine and [35,36,37,38,40,41,43,46,47,48,49,50,51,52] for povidone.

Figure 6 presents a comparison of the use of chlorhexidine and povidone for treating wound infection. The analysis included 3661 patients from 6 trials in the chlorhexidine group and 4797 patients from 14 trials in the povidone group. The Mantel–Haenszel Odds ratio was 0.6 (95% CI 0.42–0.98) and the test of heterogeneity value was especially notable at 9.28, *p* = 0.098, for the chlorhexidine group. The Mantel–Haenszel Odds ratio was 0.59 (95% CI = 0.42–0.82) and the test of heterogeneity value was of 9.39, *p* = 0.743, in the povidone group. The I^2^ values for wound infection were 46.1% in the chlorhexidine group and 0.0% in the povidone group (Table 2).

## 4. Discussion

We conducted an updated meta-analysis to evaluate the efficacy of vaginal cleansing before cesarean section in reducing postoperative endometritis. Our analysis included 31 randomized controlled trials encompassing 13,627 cesarean sections. Vaginal preparation was found to significantly decrease the risks of endometritis and postoperative fever. Specifically, povidone- and chlorhexidine-based disinfectants notably decreased the likelihood of endometritis. In addition, maternal fever incidence decreased when povidone disinfectants were used; however, a smaller effect was shown in the chlorhexidine group that in the control group. The decreased endometritis, maternal fever, and wound infection incidence indicate that preoperative vaginal cleansing should routinely be adjunct to antibiotic prophylaxis for cesarean section. These findings support the consideration of vaginal antiseptic cleansing as part of a hospital’s perioperative care. In accordance with earlier meta-analyses [16,55,56], our findings indicate reduced post-cesarean infection after vaginal cleansing [16,55,56]. Caissutti et al. [16] demonstrated that vaginal cleansing is effective in decreasing postoperative endometritis, particularly in women who are in labor or have ruptured membranes. Roeckner et al.’s findings showed a reduced rate of endometritis after the application of iodine solution in 22 randomized controlled trials (RCTs) [55]. In 2020, the Cochrane Systematic Review analyzed 20 trials and found that preoperative vaginal treatment with antiseptic solutions decreased the occurrence of post-cesarean endometritis from 7.1% to 3.1% [11]. Both iodine- and chlorhexidine-based solutions were effective in this reduction [11]. A recent meta-analysis by Liu et al. including 23 trials with 10,026 cesarean delivery patients concluded that preoperative vaginal preparation can significantly reduce the risk of post-cesarean infectious diseases (endometritis, postoperative fever, and wound infection); the results produced by 1% povidone vaginal solution were especially significant [56].

Our meta-analysis did not find a significant reduction in wound infection after vaginal cleansing, while Roeckner et al. and Liu et al. discovered a statistically significant decrease in wound infection with only the use of povidone solution [55,56]. The effectiveness of cetrimide and metronidazole as vaginal disinfectants before cesarean delivery shows varying results and a significant absence of comprehensive information. One RCT found that using cetrimide as an antiseptic for vaginal cleansing before a cesarean section reduced postpartum morbidities like fever and endometritis but did not decrease postoperative wound infections [54]. However, the data is limited, as the analysis only included one trial with 200 women [54]. In the case of metronidazole, a study involving a total of 224 patients found that of those who had received metronidazole, 7% developed post-cesarean endometritis, compared 17% of those who had received the placebo gel [53]. Administering 5 g of intravaginal metronidazole gel before surgery may decrease the occurrence of post-cesarean endometritis. This trial including only 224 women showed an OR of 1.70 for metronidazole gel in preventing wound infection [53]. Finally, a cost analysis of possible approaches applied in clinical practice for vaginal cleansing reported that the application of povidone iodine or chlorhexidine represented a low-cost intervention, and it appears to be applicable in all clinical settings [57].

The prevalence of scientific articles related to vaginal cleansing and the prevention of post-cesarean endometritis has increased substantially in recent years. The interest in this topic can be ascribed to various elements, including the cost-efficiency and effectiveness of pre-operative vaginal cleansing. The first strength of our meta-analysis is related to the number of patients included (13,627 patients) and the high number of RTCs included compared to the most recent meta-analysis by Liu et al. [56]. Second, we conducted an extensive systematic review of the literature, including the gray literature, to reduce selection bias. Third, we conducted a comprehensive analysis that included not only post-cesarean endometritis. Our meta-analysis has different limitations that mainly result from the difficulties in handling potential confounding variables and selection biases that are present in the included RCTs. Another significant limitation is the absence of stratification in subgroup analysis. Several trials involved both intrapartum and elective cesarean births, including events with ruptured and intact membranes, without specific subgroup analyses. The absence of stratification may obscure significant variations in outcomes across these various clinical settings. In addition, the lack of adjunctive data, such as serological markers, limits the accuracy of the outcome evaluation. The majority of the RCTs included in this meta-analysis did not compare multiple treatments, which represents a limitation. In addition, the socio-economic status of pregnant patients, the characteristics of the population under investigation, and the technique of placental removal, in addition to the timing and types of antibiotics administered, exhibited variation among the included studies. This variability may have a substantial impact on the results of the meta-analysis. Finally, the current comprehension of the vaginal cleansing effect is significantly limited by the absence of comprehensive research on the microbiome, specifically how it is altered after cleansing. It has been hypothesized that vaginal cleansing decreases vaginal bacteria, which reduces postoperative endometritis incidence. Nevertheless, the overall impact on the vaginal microbiota remains partially unknown. Additional research to examine the vaginal microbiome following vaginal cleaning is needed. This research is crucial for comprehending the complete range of its effects, including any possible long-term repercussions on the vaginal flora. Insufficient data necessitates a thorough evaluation of the benefits and drawbacks of vaginal cleansing prior to cesarean delivery.

## 5. Conclusions

This meta-analysis comprehensively examined the efficacy of combining vaginal cleansing with prophylactic antibiotics in the prevention of post-cesarean endometritis. The integration of data from multiple randomized trials provided a robust framework for assessing this intervention. Our findings underscore the necessity for further investigation across all vaginal disinfectant solution and procedures that can reduce post-cesarean infective complications.

## Figures and Tables

**Table 1:** Main characteristics of trials included in meta-analysis.

Study	Year	Country	No of Participants (Study vs. Control)	Intervention	Control	Primary Outcomes
Ahmed et al. [25]	2012	Egypt	218 (109 vs. 109)	0.25% chlorhexidine wipes	No vaginal cleansing	Endometritis, febrile morbidity, wound infection
Rouse et al. [26]	1997	United States	120 (62 vs. 58)	0.2% chlorhexidine solution	Sterile water	Chorioamnionitis, endometritis
Sweeten [5]	1997	United States	64 (32 vs. 32)	0.4% chlorhexidine	Sterile water	Intramniotic infection
Rouse et al. [27]	2003	United States	208 (110 vs. 98)	0.2% chlorhexidine	Sterile saline solution	Chorioamnionitis, endometritis
Lakhi et al. [28]	2019	United States	1114 (524 vs. 590)	4% chlorhexidinegluconate	10% povidone iodine	Wound infection
Hodgetts-Morton et al. [29]	2019	UK	320 (169 vs. 161)	0.05% chlorhexidine	No vaginal cleansing	Endometritis
Nnagbo et al. [30]	2022	Nigeria	300 (156 vs. 144)	Chlorhexidine solution	No vaginal cleansing	Endometritis
Urena et al. [31]	2022	Central America	204 (197 vs. 107)	4% chlorhexidine solution	Sterile saline solution	Maternal fever, endometritis
Ogah CO et al. [32]	2021	Nigeria	302 (150 vs. 152)	1.0% chlorhexidine gluconate	No vaginal cleansing	Endometritis, wound infection
Kumari et al. [33]	2023	India	760 (380 vs. 380)	7.5% chlorhexidine *w*/*v* and 15% cetrimide	No vaginal cleansing	Maternal fever, endometritis, wound infection
Haas et al. [34]	2010	United States	300 (155 vs. 145)	Povidone iodine 1%	No vaginal wash	Postoperative fever, endometritis, sepsis, readmission, wound infection, and complication
Barat et al. [35]	2016	Iran	400 (200 vs. 200)	Povidone iodine 10%	No vaginal cleansing	Postoperative fever, wound infection, endometritis
Guzman et al. [36]	2002	United States	160 (80 vs. 80)	Povidone iodine	Saline solution preparation	Endometritis, wound infection
Hassan Khedr and Fadel [37]	2016	Egypt	150 (50 vs. 100)	Povidone iodine 10%	Saline solution preparation	Endometritis
Kiani et al. [38]	2018	Pakistan	434 (217 vs. 217)	Povidone iodine 10%	Vulvar and abdominal scrubbing	Endometritis, fever, wound infection
Memon et al. [39]	2011	Pakistan	200 (100 vs. 100)	Povidone iodine 10%	No vaginal cleansing	Endometritis, fever, wound infection
Karanja David Mwangi et al. [40]	2017	Kenya	402 (206 vs. 196)	Povidone	No vaginal cleansing	Endometritis
Nandi et al. [41]	2015	India	274 (136 vs. 138)	Povidone iodine 5%	No vaginal cleansing	Endometritis, wound infection
Reid VC et al. [42]	2001	United States	430 (217 vs. 213)	Povidone iodine 10%	No vaginal cleansing	Endometritis, fever, wound infection
Starr et al. [43]	2005	United States	308 (142 vs. 166)	Povidone iodine 5%	No vaginal cleansing	Endometritis, fever, wound infection
Yildirim et al. [44]	2012	Turkey	669 (334 vs. 335)	Povidone iodine 10%	No vaginal cleansing	Endometritis
Olmez et al. [45]	2013	Turkey	667 (332 vs. 335)	Povidone iodine 5%	No vaginal preparation	Wound infection, endometritis
Tewfik et al [46]	2015	Egypt	93 (46 vs. 47)	Povidone iodine 10%	Chlorhexidine gluconate	Endometritis, fever
Charoenviboonphan [47]	2011	Thailand	599 (299 vs. 300)	Povidone iodine 1%	No vaginal painting	Combination of postoperative fever, endometritis, wound infection, and hospital length of stay
Asghania et al. [48]	2011	Iran	568 (284 vs. 284)	Povidone iodine 10%	No vaginal cleansing	Endometritis, febrile morbidity, wound infection
Kanza Gul [51]	2021	Turkey	180 (60 vs. 60 vs. 60)	Povidone iodine or saline solution	No vaginal cleansing	Maternal fever, endometritis
Tabasum Abro et al. [49]	2022	Pakistan	336 (168 vs. 168)	Povidone iodine	No vaginal cleansing	Endometritis, fever, wound infection
Abrar et al. [50]	2023	Pakistan	400 (200 vs. 200)	10% povidone iodine	No vaginal cleansing	Endometritis, fever, wound infection
Temming et al. [52]	2023	United States	608 (304 vs. 304)	1% povidone-iodine	No vaginal cleansing	Superficial or deep surgical-site infection, endometritis, fever
Pitt et al. [53]	2001	United States	224 (112 vs. 112)	Metronidazole 5 g 0.75% gel	Placebo vaginal gel	Endometritis, fever, wound infection
Mohamed et al. [54]	2015	Egypt	200 (100 vs. 100)	Cetrimide	No vaginal cleansing	Endometritis, fever, wound infection

**Table 2:** Outcomes analyzed.

	Endometritis			Maternal Fever			Wound Infection		
Solution	Studies/ Women, n	OR-I^2^ (%)	95% CI	Studies/ Women, n	OR-I^2^ (%)	95% CI	Studies/ Women, n	OR-I^2^ (%)	95% CI
Chlorhexidine	10/6230	0.56 *–47.3	0.45–0.70	5/2598	0.52–0	0.34–0.79	5/2429	0.65–46.1	0.45–0.94
Povidone	19/7173	0.47 *–54.5	0.38–0.60	18/6965	0.47 *–70.5	0.38–0.57	14/4797	0.59–0	0.42–0.82

* *p*-value < 0.05.

## Data Availability

Data sharing is not applicable to this article as no new data were created or analyzed in this study.

## Abbreviations

The following abbreviations are used in this manuscript:

CS	Cesarean Section
RCT	Randomized Controlled Trial
PICO	Population, Intervention, Control, Outcome
OR	Odds Ratio
CI	Confidence Interval

## Appendix A

**Table A1:** Detailed search strategy for systematic review.

Variable	Search Strategy
Database searched	MEDLINE (accessed through PubMed), Scopus, EMBASE, Google scholar and Cochrane Central Register of Controlled Trials (CENTRAL) from their origin until 23 January 2024
Search strategy for Pubmed	((((((Caesarean delivery) OR (cesarean section)) AND (endometritis)) OR (postoperative infection)) OR (fever)) AND (vaginal preparation)) OR (vaginal cleansing)
Scopus	((((((caesarean delivery OR cesarean section) AND endometritis) OR postoperative infection) OR fever) AND vaginal preparation) OR vaginal cleansing)
CENTRAL	((((((caesarean delivery OR cesarean section) AND endometritis) OR postoperative infection) OR fever) AND vaginal preparation) OR vaginal cleansing)
Google scholar	(“caesarean delivery” OR “cesarean section”) AND (“vaginal preparation” OR “vaginal cleansing”) AND (endometritis OR “postoperative infection” OR fever)
EMBASE	(‘caesarean delivery’/exp OR ‘cesarean section’/exp) AND (‘vaginal preparation’/exp OR ‘vaginal cleansing’/exp) AND (‘endometritis’/exp OR ‘postoperative infection’/exp OR fever/exp)
Other sources	The reference lists of selected articles and reviews were hand searched to identify any relevant articles.
